# Case Report: Diagnostic utility of pleural tissue debris retrieved during percutaneous drainage for the diagnosis of malignant pleural effusion and molecular profiling

**DOI:** 10.3389/fonc.2026.1967294

**Published:** 2026-10-06

**Authors:** Jiwon Kim, Han Gyeol Kim, Seung Hyeun Lee

**Affiliations:** 1 Division of Pulmonary, Allergy, and Critical Care Medicine, Department of Internal Medicine, Kyung Hee University Hospital, College of Medicine, Kyung Hee University, Seoul, Republic of Korea; 2 Department of Pathology, Kyung Hee University Hospital, College of Medicine, Kyung Hee University, Seoul, Republic of Korea; 3 Department of Precision Medicine, Graduate School, Kyung Hee University, Seoul, Republic of Korea

**Keywords:** effusion drainage, immunohistochemistry, lung cancer, molecular profiling, pleural tissue debris

## Abstract

**Background:**

Although pleural tissue debris is occasionally encountered in the drainage systems of patients undergoing catheter or chest tube drainage, its clinical utility has not been reported. Here, we report two cases in which pleural tissue debris retrieved during catheter drainage enabled a definitive diagnosis of malignant pleural effusion (MPE) and molecularly guided treatment decisions.

**Case presentation:**

The first case involved a 67-year-old woman with rheumatoid arthritis-associated interstitial lung disease who developed a right pleural effusion suggestive of lung cancer with pleural metastasis. Invasive procedures for tissue acquisition were required; however, biopsy was withheld because of the high risk of pneumothorax and acute exacerbation of the underlying lung disease. Tissue debris was incidentally found in the drainage system. The tissue was adequate for pathological examination and revealed lung squamous cell carcinoma without genomic alterations. Chemoimmunotherapy was initiated without further invasive procedures. The second case involved a 78-year-old woman who presented with a left pleural effusion and a brain lesion, highly suggestive of lung cancer with brain metastasis. She declined invasive diagnostic procedures; however, tissue debris was incidentally collected from the drainage bottle and diagnosed as lung adenocarcinoma. Molecular testing revealed that the tumor harbored an epidermal growth factor receptor (EGFR) L858R mutation, allowing first-line targeted therapy to be initiated without further invasive testing.

**Conclusion:**

To the best of our knowledge, this is the first case report to describe the clinical utility of pleural tissue debris for the diagnosis of MPE and its suitability for molecular profiling. Our report highlights that this previously overlooked byproduct of routine pleural drainage can provide adequate material for histopathological diagnosis, immunohistochemistry, and molecular testing in patients with MPE, particularly those for whom conventional biopsy is contraindicated or declined.

## 1 Introduction

Despite advances in cytopathological techniques and extensive research on various biomarkers, the diagnosis of malignant pleural effusion (MPE) remains challenging in clinical practice. Cytopathological detection of malignant cells in pleural effusion remains the gold standard for the diagnosis of MPE (1). However, this approach has limited sensitivity, thus repeated fluid collection or larger volumes are often required to improve diagnostic performance (2). When cytology is negative but clinical suspicion of malignancy persists, tissue confirmation through needle or thoracoscopic pleural biopsy is necessary. These procedures carry meaningful risks, including pneumothorax rates of up to 15% for transthoracic needle biopsy and a pooled complication rate of approximately 4% for thoracoscopy (3, 4). In addition, for older patients or those with significant comorbidities, these risks may be prohibitive, leaving a persistent gap in the availability of a diagnostic strategy that is both accurate and avoids the need for an additional invasive procedure.

During catheter or chest tube drainage for pleural effusion, fragments of pleural tissue are occasionally found within the drainage system; however, the clinical relevance of this incidentally obtained material has scarcely been characterized. Here, we describe two patients with lung cancer-associated MPE in whom the diagnosis was established using pleural “tissue debris” retrieved from the drainage apparatus. In both cases, the retrieved specimen was sufficient for molecular testing, including analysis of epidermal growth factor receptor (EGFR) mutations and anaplastic lymphoma kinase (ALK) rearrangements, as well as immunohistochemical evaluation of programmed death-ligand 1 (PD-L1) expression, allowing systemic therapy to be initiated without additional invasive procedures. These cases suggest that this previously overlooked, incidentally captured specimen may represent a practical, minimally-invasive diagnostic resource for the diagnosis of MPE and merits systematic surveillance during routine effusion management.

## 2 Case presentations

### 2.1 Case 1

A 67-year-old female was referred to the Department of Pulmonology with worsening dyspnea and chest imaging abnormalities. She had been treated for rheumatoid arthritis (RA) for seven years at another hospital and was taking tacrolimus 2 mg daily, methylprednisolone 4 mg daily, and methotrexate 15 mg weekly. She had a 30 pack-year smoking history and had quit smoking 1 year prior. Chest computed tomography (CT) scan performed at presentation showed pleural effusion and several pleural nodules (arrows) in the right hemithorax (**Figure 1A**). In addition, the lung window on chest CT showed honeycomb changes predominantly in the basal and peripheral regions of both lungs, consistent with RA-related usual interstitial pneumonia (UIP) (**Figure 1B**). Given her dyspnea and the anticipated need for continued drainage, a pigtail catheter—rather than a single thoracocentesis—was inserted for symptomatic management, and analysis of the pleural effusion revealed a lymphocyte-dominant exudate with a low adenosine deaminase (ADA) level (12.7 IU/L). Cytology with cell block preparation was negative on two consecutive tests. Transthoracic echocardiography revealed impaired relaxation with increased left ventricular filling pressure, suggesting diastolic heart failure. Pulmonary function test showed a forced vital capacity of 59% of the predicted value, a forced expiratory volume in 1 s of 59% of the predicted value, and a diffusion capacity of 51% of the predicted value, consistent with restrictive ventilatory impairment due to the underlying UIP. Because the pleural nodularity on chest CT was atypical for an effusion related solely to RA-associated pleuritis, which more commonly manifests as diffuse pleural thickening rather than discrete nodules, malignancy was specifically suspected rather than assessed as part of routine screening. ^18^F-fluorodeoxyglucose positron emission tomography (FDG PET) showed an approximately 15 mm hypermetabolic nodule in the superior segment of the right lower lobe and diffuse hypermetabolism along the pleura in the right hemithorax, indicative of lung cancer with pleural metastasis (**Figure 1C**). Because cytology revealed negative results, pathological confirmation was necessary; however, invasive procedures, including percutaneous needle biopsy of the lung nodule, could not be performed because of the potential risk of procedure-related pneumothorax. We counseled the patient and her family regarding video-assisted thoracoscopic (VAT) pleural biopsy under general anesthesia; however, they were unable to decide whether to undergo the procedure because of the potential risk of acute exacerbation of the underlying UIP after surgery. While they considered their decision, we initiated low dose pirfenidone (200 mg three times daily) and planned to increase the dose if she did not experience significant adverse events, including photosensitivity and gastrointestinal symptoms. Pirfenidone was administered as a bridging antifibrotic measure aimed at reducing the risk of acute UIP exacerbation while the diagnostic decision was being made. On hospital day 4, during routine morning rounds, we incidentally observed a tissue debris-a pinkish, lump-like soft-tissue fragment measuring approximately 15 mm in greatest dimension- in a transparent extension tube (**Figure 1D**). Therefore, we immediately collected the samples (**Figure 1E**), secured them in formalin bottles, and sent them to the pathology department. Pathological examination revealed squamous cell carcinoma (SQCC), with strong positivity for p40 on immunohistochemical (IHC) staining (**Figures 1F, G**). Using this tissue, we performed molecular testing for EGFR using real-time PCR assay (PANAMutyper™, PANAGENE, Seoul, South Korea) and IHC for ALK using an anti-ALK antibody (clone D5F3) and an automated VENTANA BenchMark ULTRA instrument (Ventana Medical Systems, Inc., Tucson, AZ, USA). We also assessed the tumor proportion score for PD-L1 using PD-L1 IHC 22C3 pharmDx assay (Agilent Technologies, Inc., Carpinteria, CA, USA). No genetic alterations were detected and PD-L1 expression was negative. Further staging workup revealed no metastases to other organs; thus, a final diagnosis of stage IVA lung SQCC with right pleural metastasis was made. Based on these results, we initiated first-line treatment with pembrolizumab and paclitaxel/carboplatin. Through the third cycle of treatment, the patient tolerated the regimen except for grade 2 alopecia, grade 1 neutropenia, and grade 1 peripheral neuropathy, which were readily managed with dose reductions of the cytotoxic agents. No exacerbation of the underlying RA or UIP was observed.

**Figure 1:**
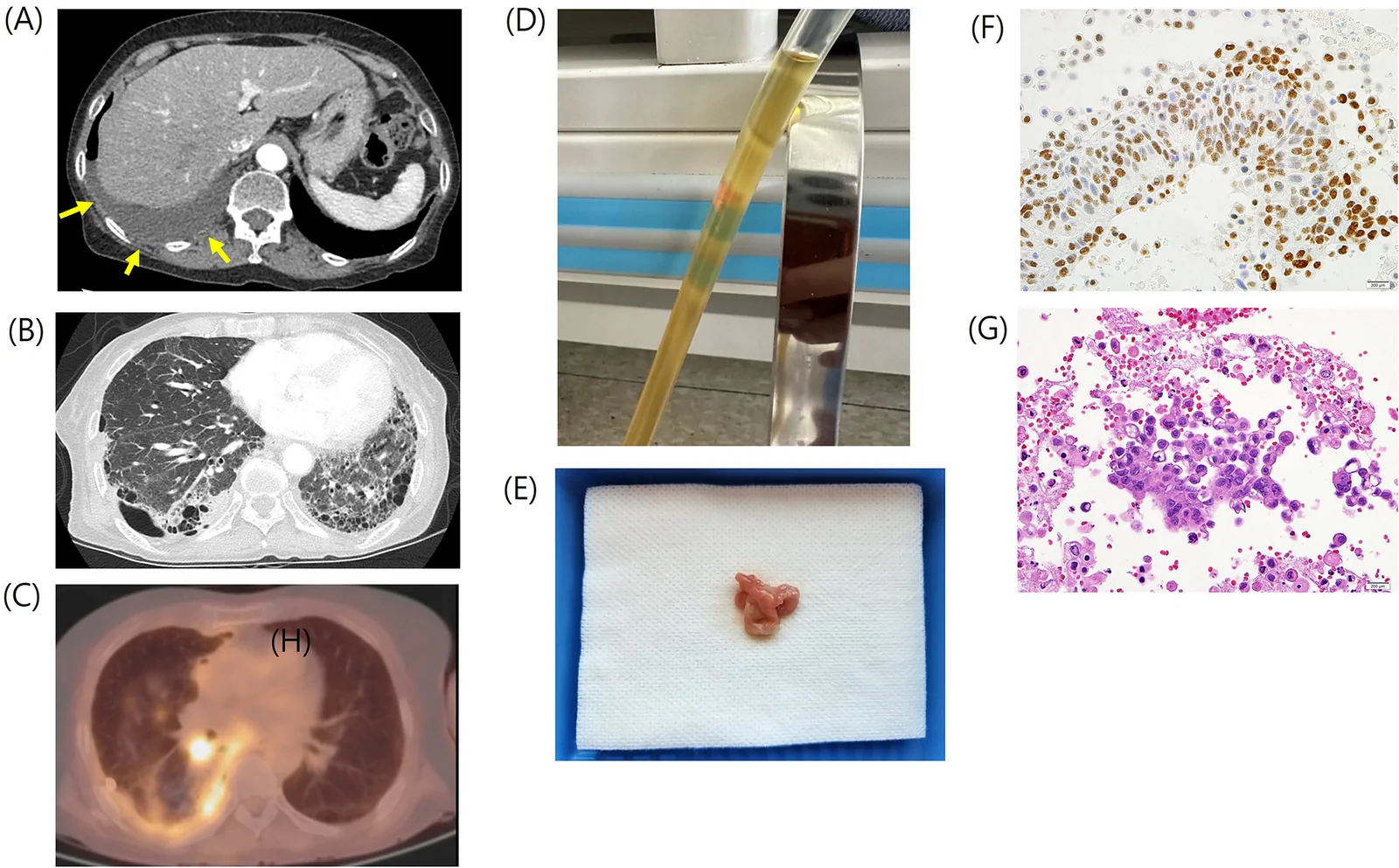
Clinical and pathological findings of Case 1. **(A)** Contrast-enhanced chest CT scan showing a right-sided pleural effusion with multiple pleural nodules (arrows). **(B)** In the lung window setting, honeycombing predominantly seen in both basal lungs compatible with usual interstitial pneumonia. **(C)** FDG PET scan revealing diffuse hypermetabolism in the right pleura, suggestive of pleural metastasis. **(D, E)** Tissue debris collected from the drainage extension tube and its gross finding. **(F)** Microscopic findings of the tissue debris showing nests of squamous cell carcinoma within a mesothelial background (H&E stain, ×400). **(G)** Immunohistochemical staining for p40 showing strong nuclear expression in the tumor cells (×400).

### 2.2 Case 2

A 78-year-old female presented to our department with one-month history of cough. She had never smoked and was taking metformin for diabetes. Chest CT (**Figure 2A**) revealed diffuse pleural thickening and pleural effusion in the left hemithorax. Given her persistent cough and the anticipated need for continued drainage, a pigtail catheter—rather than a single thoracocentesis—was inserted for symptomatic management, and analysis of pleural effusion revealed a lymphocyte-dominant exudate with a low ADA level (23.8 IU/L). Cytology of a cell block was negative for malignancy on two occasions. FDG PET revealed a hypermetabolic mass in the left apical lung (**Figure 2B**), and brain magnetic resonance imaging (MRI) showed an enhanced nodule in the left occipital lobe (**Figures 2C**, arrowhead), which was highly suggestive of lung cancer or pleural mesothelioma with brain metastasis. However, she declined further invasive procedures, despite her caregivers’ preference for the necessary diagnostic procedures and systemic treatment, if indicated. On hospital day 3, during routine rounding, we unexpectedly observed a reddish, lump-like soft-tissue fragment measuring approximately 25 mm in greatest dimension floating in the chest drainage bottle (**Figures 2D**, arrow); thus, we collected the sample (**Figure 2E**) and delivered it to the pathology department, as described above. Pathological examination revealed adenocarcinoma in a mesothelial background (**Figure 2F**), with strongly positivity for thyroid transcription factor 1 (**Figure 2G**), consistent with lung adenocarcinoma (LUAD). Using the tissue debris, we performed the aforementioned molecular testing using the same platforms as the previous case and found that the tumor harbored an EGFR L858R mutation (**Figure 2H**). Based on these results, we initiated first-line treatment with lazertinib, a third-generation EGFR-tyrosine kinase inhibitor, without further invasive procedures. To date, she has tolerated this regimen with some adverse events, including stomatitis, acneiform rash, and peripheral neuropathy, all of which were grade 2 or lower. The tumor responded well, and she has continued treatment for 8 months.

**Figure 2:**
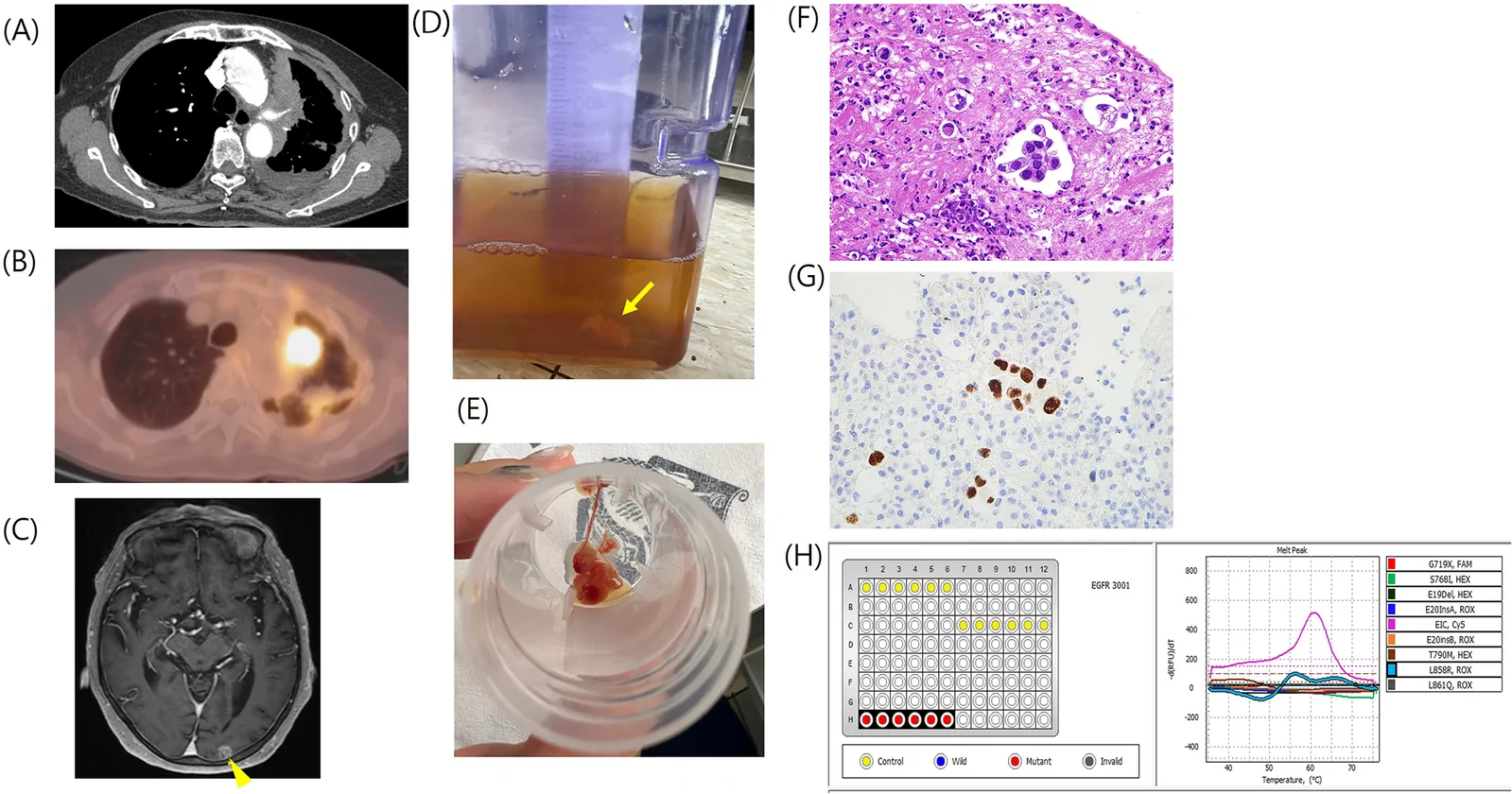
Clinical and pathological findings of Case 2. **(A)** Contrast-enhanced chest CT scan showing a left-sided pleural effusion with associated pleural thickening. **(B)** FDG PET scan revealing intense hypermetabolism in the left pleura and lung parenchyma, suggestive of malignant involvement. **(C)** Brain MRI demonstrating a small enhancing metastatic lesion in the left occipital lobe (arrow head). **(D)** Gross photograph of the amber-colored pleural fluid collected in a drainage bottle (arrow). **(E)** Reddish, stringy tissue debris detected and collected from the pleural fluid. **(F)** Pathological examination reveals nests of adenocarcinoma cells within a fibrinous and inflammatory background (H&E stain, ×400). **(G)** Immunohistochemical staining for TTF-1 showing strong nuclear positivity in the tumor cells (×400). **(H)** EGFR mutation analysis using the PANAMutyper™ PCR assay. Melting peak analysis showing a distinct peak corresponding to the L858R mutation (cyan line) at the specific melting temperature, with the internal control (pink line), confirming the presence of an EGFR L858R mutation.

## 3 Discussion

Our cases demonstrate that pleural tissue debris, an incidental specimen recovered during routine catheter or chest tube drainage, can provide a definitive diagnostic alternative when conventional biopsy is not feasible in patients with suspected MPE. Notably, in both instances, the retrieved fragments were sufficient for comprehensive ancillary testing, including EGFR mutation analysis and IHC for ALK and PD-L1. These results directly guided first-line systemic treatment selection. Consequently, both patients were able to initiate molecularly tailored therapy without undergoing further invasive procedures. To the best of our knowledge, this case report is among the first to demonstrate that pleural tissue debris can establish a definitive diagnosis of MPE while simultaneously supporting comprehensive molecular profiling to guide individualized treatment decisions.

The mechanism underlying the spontaneous appearance of pleural tissue fragments in the drainage system has not yet been established. One plausible explanation is a procedure-related process in which catheter insertion mechanically disrupts the pleural surface, dislodging tissue fragments that are subsequently evacuated together with the effusion. Alternatively, such fragments may have been present as free-floating material within the pleural space before catheterization, having detached spontaneously as a consequence of the underlying disease process rather than the procedure itself. A biology-driven mechanism may also be relevant: reduced intercellular cohesion in neoplastic tissue, for example via epithelial-mesenchymal transition-associated loss of E-cadherin-dependent adherens junctions, has been proposed to facilitate detachment of tumor cell clusters from the primary site (5, 6). This remains a hypothesis for future mechanistic investigation rather than an established explanation, and a two-case series cannot determine whether malignant tissue is intrinsically more susceptible to exfoliation than tissue affected by benign processes (7). Because the present report was limited to two cases, we were unable to determine the relative contribution of these mechanisms. Larger clinical studies and dedicated mechanistic studies are needed to clarify how and why pleural tissue can be spontaneously released into the drainage circuit.

Although these two cases demonstrate that pleural tissue debris can yield a definitive diagnosis of MPE, whether spontaneous tissue shedding and its diagnostic utility occurs across other etiologies of pleural effusion remains uncertain. In tuberculous (TB) pleurisy, ADA is widely used as a surrogate biomarker; however, its diagnostic sensitivity remains suboptimal, and confirmatory pleural biopsy is still required in a subset of cases (8). In principle, tissue debris could have diagnostic utility in TB pleurisy if granulomatous tissue is similarly shed into the drainage system. However, previous studies have demonstrated that granuloma-associated macrophages undergo epithelialization, marked by the upregulation of E-cadherin and other junctional proteins, and become encapsulated within a fibrotic cuff. This configuration contrasts with the loss of intercellular adhesion characteristic of malignant tissues (9, 10). This structural stability suggests that granulomatous tissues are comparatively resistant to spontaneous exfoliation, implying that both the frequency and diagnostic yield of shed tissue debris may be lower in TB pleurisy than in MPE. Further studies involving diverse etiologies are therefore needed to define the overall frequency of tissue shedding across different types of pleural effusion and to identify the clinical and procedural factors that influence its occurrence.

Another notable observation concerns the adequacy of pleural tissue debris for ancillary pathological testing. Biomarker assessment, including IHC evaluation of predictive markers, such as PD-L1, and molecular profiling for actionable driver alterations, is central to treatment selection in advanced lung cancer (11). We defined specimen adequacy operationally as the presence of sufficient tumor tissue on hematoxylin and eosin (H&E)-stained sections to permit at least one lineage-defining immunostain together with completion of the full companion molecular panel without the need for repeat sampling; this criterion was met in both cases described here. In both cases, the retrieved debris was sufficient not only for histologic subtyping, but also for a full molecular and IHC testing enabling first-line treatment selection. Because tissue debris consists of macroscopic, three-dimensional tumor fragments rather than dispersed cytological cells, it may hypothetically offer specimen quantity and quality comparable to those obtained by conventional pleural biopsy; however, this equivalence is unproven and would require confirmation in a larger, systematically collected series with direct comparison to biopsy specimens. Taken together, these two cases suggest that, when retrievable, tissue debris can support both definitive histopathological diagnosis and the ancillary testing required for precision oncology without the need for an additional invasive procedure. In both cases, a primary lung malignancy was favored over mesothelioma on the basis of strong lineage-specific IHC positivity (p40 in Case 1, TTF-1 in Case 2), neither of which is expressed by mesothelioma, together with a radiologic pattern of a discrete pulmonary nodule or mass with pleural metastasis rather than the diffuse pleural rind typical of mesothelioma. A dedicated mesothelioma-exclusion panel (e.g., calretinin, WT1, D2-40, BAP1) was not performed in either case, and this should be regarded as a limitation rather than evidence of an exhaustive differential diagnosis. There was no pre-specified size or color threshold for specimen retrieval; any macroscopically visible, discrete fragment distinguishable from blood clot or fibrinous material was collected. In our cases the retrieved fragments were tan-to-whitish (Case 1, **Figure 1E**) and reddish and stringy (Case 2, **Figure 2E**) in gross appearance. A grossly hemorrhagic specimen without a discernible solid component would be unlikely to yield diagnostic material; systematic documentation of debris size, color, and consistency should be incorporated into future studies.

Although, pleural fluid cytology is the standard diagnostic test for MPE, it has an inconsistent and often suboptimal sensitivity. A negative result does not exclude malignancy, and repeated sampling provides diminishing diagnostic yield, with sensitivity falling to approximately 22% for the second specimen and 14% for the third (12, 13). Reported sensitivity ranges from 46% to 60% across large cohorts, and a meta-analysis of 36 studies involving 6,057 patients estimated a pooled sensitivity of 58.2%, with substantially lower diagnostic yields for squamous cell carcinoma and mesothelioma than for adenocarcinoma (14). These limitations frequently necessitate additional invasive testing, which contributes to diagnostic delays. To address these limitations of cytology, numerous biomarkers have been investigated for the diagnosis of MPE; however, none have been sufficiently validated for incorporation into routine clinical practice. More recently, methylation profiling of cell-free DNA (cfDNA) in pleural fluid has been explored as means of discriminating malignant from benign effusions, and genomic interrogation of effusion-derived cfDNA has likewise shown promise. In one prospective cohort of 50 patients with newly diagnosed advanced NSCLC and malignant effusion, actionable mutations were detected in 88% of cases using effusion cfDNA, representing a substantially higher yield than that obtained from the cell pellet (78%) or tumor tissue (66%) (p = 0.031) (15). Despite this diagnostic potential, cfDNA-based sequencing has important limitations: because it provides genotypic information without tissue morphology, it cannot independently establish a pathological diagnosis, determine tumor lineage, or identify histological transformation events, such as small cell transformation following targeted therapy, nor can it support protein-based assays, such as IHC for PD-L1 expression. Tissue debris may overcome these constraints by preserving an intact tissue architecture suitable for histopathological evaluation, immunohistochemistry, and molecular testing within a single specimen. This was demonstrated in our two patients, both of whom had negative cytology with cell blocks but received definitive diagnosis based on tissue debris. Rather than competing modalities, tissue debris- and effusion-based cfDNA analyses may ultimately prove complementary. Future work should define how these two specimens, each obtainable without an additional invasive procedure, can best be integrated into the diagnostic pathway for MPE.

In conclusion, our cases illustrate that pleural tissue debris, an incidental by-product of routine pleural effusion drainage, can provide a definitive histopathological diagnosis and support comprehensive molecular and IHC profiling. It may be particularly valuable in several recurring clinical scenarios. First, it can help resolve cases in which pleural fluid cytology yields an indeterminate result, such as “atypical cells present” or “suspicious for malignancy,” which falls short of a definitive diagnostic threshold. Second, it can serve as an additional tissue source when ancillary testing requires more material than the primary specimen can provide. Third, and perhaps most clinically important, it offers a diagnostic avenue in patients for whom invasive procedures are not feasible, including those unable to discontinue antiplatelet or anticoagulant therapy, those who decline biopsy, and those of advanced age or poor performance status. Overall, our report provide proof-of-concept evidence that systematic attention to this previously overlooked material may expand the diagnostic options available for pleural effusion. Prospective, adequately powered, multicenter studies are warranted to validate the diagnostic yield of pleural tissue debris across diverse effusion etiologies, standardize its collection and handling, and ultimately define its role as a minimally invasive adjunct that avoids the need for further invasive tissue sampling in the diagnostic evaluation of pleural effusion.

## Data Availability

The raw data supporting the conclusions of this article will be made available by the authors, without undue reservation.
